# Inflammatory and Other Biomarkers: Role in Pathophysiology and Prediction of Gestational Diabetes Mellitus

**DOI:** 10.3390/ijms160613442

**Published:** 2015-06-11

**Authors:** Sally K. Abell, Barbora De Courten, Jacqueline A. Boyle, Helena J. Teede

**Affiliations:** 1 Monash Centre for Health Research and Implementation, School of Public Health and Preventive Medicine, Monash University, Clayton 3168, Australia; E-Mails: sally.abell@monash.edu (S.K.A.); barbora.decourten@monash.edu (B.D.C.); jacqueline.boyle@monash.edu (J.A.B.); 2 Diabetes and Vascular Medicine Unit, Monash Health, Clayton 3168, Australia; 3 Monash Women’s Services, Monash Health, Clayton 3168, Australia

**Keywords:** biomarkers, inflammatory markers, pregnancy, gestational diabetes mellitus, risk prediction

## Abstract

Understanding pathophysiology and identifying mothers at risk of major pregnancy complications is vital to effective prevention and optimal management. However, in current antenatal care, understanding of pathophysiology of complications is limited. In gestational diabetes mellitus (GDM), risk prediction is mostly based on maternal history and clinical risk factors and may not optimally identify high risk pregnancies. Hence, universal screening is widely recommended. Here, we will explore the literature on GDM and biomarkers including inflammatory markers, adipokines, endothelial function and lipids to advance understanding of pathophysiology and explore risk prediction, with a goal to guide prevention and treatment of GDM.

## 1. Introduction

In developed nations, antenatal care for the pregnant woman usually starts in the first trimester, with subsequent visits approximately monthly until 28 weeks, then increasing in frequency with fortnightly visits to 36 weeks, and weekly until delivery [1]. In this model, the greater proportion of visits in the third trimester aligns with increased complications in the later stages of pregnancy. Ideally, these complications need to be predicted in the first and second trimesters to enable prevention [1]. Pregnancies are currently classified as “low” or “high” risk based on the likelihood of an adverse maternal or neonatal outcome [2]. However, it is increasingly recognised that this classification may be too simplistic and fails to adequately ascertain the spectrum of risk [2]. It has become apparent that integrated patient assessment in the first trimester using maternal history and characteristics, and biochemical tests, may better define risk for pregnancy complications including foetal abnormalities, miscarriage, stillbirth, pre-eclampsia (PE), preterm birth, gestational diabetes mellitus (GDM), intrauterine growth restriction (IUGR) and macrosomia [1]. Not only would this define patient-specific risk [1], but it would allow early commencement of preventative therapies, institution of appropriate models of antenatal care and optimal level of surveillance [1,2]. It would also allow recruitment of high risk populations to trials of interventions to develop better strategies for prevention of pregnancy complications and for improvement of maternal and foetal pregnancy outcomes [1,2].

Obesity is increasing in prevalence world-wide, and contributes significantly to risk of pregnancy complications. Obesity is a chronic inflammatory state. Pregnancy, and furthermore GDM are also associated with an increase in inflammatory markers, and thus a heightened inflammatory response may play an important role in development of pregnancy complications [3]. Current literature suggests an emerging role for the use of inflammatory and other biomarkers to improve understanding of the pathophysiology of adverse pregnancy outcomes, including the impact of obesity [3,4,5,6]. Prediction models based on maternal history and risk factors alone have variable performance for adverse outcomes such as PE, IUGR, preterm birth, GDM and macrosomia [2]. Addition of inflammatory and other biomarkers with a proven role in pathophysiology of these outcomes may be usefully incorporated into early prediction models. A good example is in prediction models for PE [7], which affects 2% of pregnancies and is a major cause of maternal and neonatal morbidity and mortality [1]. Algorithms combining maternal characteristics and biochemical tests at 11–13 weeks could potentially identify 90%, 80%, and 60% of pregnancies that are complicated by early (before 34 weeks), intermediate (34–37 weeks) and late (after 37 weeks) onset PE [1,8].

This review will discuss the current clinical dilemmas of increasing obesity and GDM, and their impact on adverse pregnancy outcomes. We will outline the pathophysiology of inflammation in obesity, pregnancy and in GDM. We will briefly outline inflammatory pathways involved in development of type 2 diabetes mellitus (T2DM). We will provide insights into the pathophysiology of GDM by exploring the literature on serum biomarkers including inflammatory markers, adipokines, endothelial function markers, and lipid metabolism. We will summarise current literature on the potential use of these markers to predict GDM, and determine the current role of predictive models for early intervention in women at high risk of GDM.

## 2. Obesity

**Figure 1:**
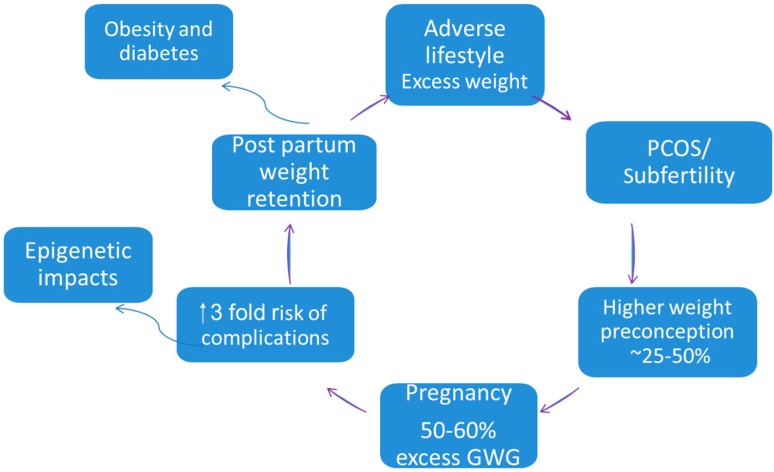
The vicious cycle of obesity and reproductive complications. Women with adverse lifestyle factors and excess weight are more likely to develop polycystic ovarian syndrome (PCOS) and subfertility. They are likely to enter pregnancy overweight and are at increased risk for excess gestational weight gain (GWG). Obesity and excess GWG results in a three-fold increased risk of adverse pregnancy outcomes including gestational diabetes mellitus (GDM), and may have epigenetic impacts including long term metabolic syndrome and cardiovascular disease for mother and baby. Post-partum weight retention contributes to prevalent obesity and type 2 diabetes mellitus (T2DM) long-term and to risks in subsequent pregnancies [10].

In both developed and developing countries, more women are obese at conception, and young reproductive women are at high risk of excess weight gain driving obesity and related reproductive and metabolic complications (see Figure 1) [9]. In Australia, trends predict a 65% increase in obesity prevalence by 2025, with <30% of women in the healthy weight range [10]. Obesity results in a ~3-fold increased risk of GDM [11], and increases antenatal risks include hypertensive disorders and thromboembolic complications [4]. Peripartum, obese women have higher rates of induction of labour, operative delivery and postpartum haemorrhage [4]. High pregravid body mass index (BMI) and excessive gestational weight gain (GWG) are both important predictors of short-term morbidity and higher weight retention postpartum [12], increased risks in future pregnancies, and long term obesity [13]. Offspring of obese mothers tend to be large for gestational age, require admission to neonatal units, have higher risks of congenital anomalies, mortality and lifetime risk of obesity and metabolic syndrome [4]. Prevention of obesity and gestational weight gain are public health priorities to break this vicious cycle.

## 3. Inflammation and Biomarkers in Obesity

Since the discovery of leptin in 1994, adipose tissue is widely regarded as an endocrine organ, capable of secreting various adipokines [14]. Adipokines are proteins secreted by adipose tissue that act as paracrine factors in adipose tissue and as endocrine hormones in liver, muscles and the central nervous system [15].

Obesity is accompanied by increased release of free fatty acids (FFAs) and altered secretion of adipokines such as leptin, adiponectin, resistin and retinol-binding protein-4 (RBP-4) from adipocytes [16,17]. In obesity, adipokines secreted by adipocytes and macrophages also act in an autocrine fashion to further exacerbate adipose tissue inflammation, and decrease muscle and liver insulin sensitivity [17]. Furthermore, altered adipokine secretion can act directly on the hypothalamus to increase food intake and reduce energy expenditure [17]. Low concentrations of adiponectin, the most abundant adipose-specific protein, occur in obesity and predict both a decrease in insulin sensitivity and development of type 2 diabetes mellitus (T2DM) [18].

The functional activity of adipose tissue macrophages is proportional to the degree of obesity. In obese individuals, there is an increase in macrophage density and a shift toward the M1 “classically activated” phenotype (probably driven by T-helper-1 (Th-1) cytokines), secreting pro-inflammatory cytokines such as tumour necrosis factor alpha (TNF-α), interleukin-6 (IL-6) and interleukin-12 (IL-12) and generating reactive oxygen species such as nitric oxide via activation of nitric oxide synthase (NOS) [4].

Cellular mechanisms responsible for enhanced macrophage recruitment in obesity are largely unknown, but increased adipocyte size and dysregulated adipokine production promote cross-talk between adipocytes and macrophages [19]. Adipocyte-derived factors such as monocyte-chemoattractant protein-1 (MCP-1) and colony stimulating factor-1 (CSF-1) are over-expressed and can promote recruitment of circulating monocytes [20]. Increased FFAs from enlarged adipocytes act as ligands for toll-like receptors (TLRs) and induce production of inflammatory cytokines from macrophages through activation of the nuclear factor-κB (NF-κB) pathway, which has been shown to be related to insulin resistance [17,21,22].

## 4. Inflammatory Pathways in Type 2 Diabetes Mellitus

Women who develop GDM in pregnancy have a much higher risk of developing T2DM post-partum. Chronic low-grade activation of the immune system (increased plasma inflammatory markers without overt signs of inflammation) has been suggested to play an aetiologic role in the development of T2DM. This is supported by cross-sectional [23,24,25,26,27,28] and prospective [25,29,30,31,32,33] studies of associations between increased markers of inflammation and obesity, insulin resistance and/or T2DM. We provided the first evidence in healthy normal glucose tolerant individuals that chronic low-grade activation of the immune system may contribute to the development of T2DM by causing a decline in insulin sensitivity [25]. Our data were confirmed by two other studies [34,35]. The exact mechanism by which activation of the immune system impairs insulin action is not completely understood. The NF-κB and I kappa B kinase (IKKβ) and c-Jun NH2-terminal kinase (JNK) pathway have been proposed as links between activation of the immune system and the development of insulin resistance and T2DM [36,37]. We have shown these two pathways are important in humans [22].

## 5. Inflammatory Pathways in Pregnancy

While most pregnancies are classified as low risk and progress normally, screening and monitoring for possible adverse outcomes for the mother and foetus are routine. These include screening for foetal aneuploidies, miscarriage and foetal death, disorders of placentation, PE, pre-term delivery, IUGR, GDM and macrosomia [38]. As discussed, obesity may worsen risk for adverse pregnancy outcomes. Recent scientific advances are focused on early prediction and screening for these complications to enable targeted prevention and therapy [1].

Pregnancy is characterized by an altered inflammatory profile compared to the non-pregnant state, with a fine balance between pro- and anti-inflammatory cytokines needed for normal development. Physiological regulation of the innate immune response and changes in cytokine production prevent rejection of the foetal allograft throughout pregnancy [3]. In addition to local T cells, non-lymphoid tissues including the placenta, and in particular trophoblast cells, are major sites of cytokine production in pregnancy [3,39]. During a normal pregnancy, the balance of T-helper cell activity is strongly shifted toward an anti-inflammatory profile characterised by Th-2 cytokines, which have a protective role in the foetal-maternal relationship and favour normal pregnancy outcomes [3]. However, infective or inflammatory processes (e.g., Obesity, GDM and other states of insulin resistance) superimposed on pregnancy may alter this balance and compromise normal development [3,40].

Immune-endocrine interactions modulate responses to environmental perturbations in pregnant women [3]. Maternal and placental hormones including progesterone, relaxin, activin A and oxytocin are involved in progression of a normal pregnancy and also have direct impact on inflammatory pathways and immune-mediated complications [3]. Hypoxia and the innate immune response are adaptive mechanisms mediated by interactions between tissue remodelling factors like matrix metalloproteinases (MMP) and vasoactive and hemostatic factors like prostaglandins and coagulation factors [3]. Toll-like receptors (TLRs), pattern recognition receptors central to the innate immune response, also appear to have a significant role in normal pregnancy [3].

Adipokines contribute to regulation of maternal energy metabolism and insulin resistance [5]. A heightened inflammatory response mediated by adipokines, both locally (adipose tissue, placenta and vascular endothelium) and systemically (circulating plasma concentrations) may be involved in adverse clinical outcomes during pregnancy [4]. Normal pregnancy-induced insulin resistance is further enhanced in pregnancy complications such as GDM, and in those associated with disturbed placental function such as PE and intrauterine growth restriction [5].

Obesity is an important contributor when studying interrelationships between inflammation and adverse pregnancy outcomes (see Figure 1). Expansion of adipose tissue leads to further enhanced macrophage recruitment and production of pro-inflammatory cytokines such as TNF-α and IL-6. Obesity and excess GWG in pregnancy also increase the risk of GDM [41].

## 6. Gestational Diabetes Mellitus (GDM)

In normal pregnancy, insulin resistance increases in the late second trimester to levels that approximate that seen in T2DM [14,42]. Most women remain normoglycaemic due to adequate beta cell compensation with higher insulin secretion [14]. However, GDM develops if beta-cell compensation is inadequate for the level of insulin resistance and hepatic glucose production [14,43].

GDM is a condition of carbohydrate intolerance with onset or first recognition in pregnancy [44]. The incidence of GDM is increasing in line with advanced maternal age and the obesity epidemic [45]. A follow-up publication to the hyperglycaemia and adverse pregnancy outcomes (HAPO) study found that the frequency of GDM among their 15 collaborating centres was 17.8% (range 9.3%–25.5%) using International Association of Diabetes and Pregnancy Study Group (IADPSG) criteria [46]. GDM is associated with adverse maternal health outcomes such as gestational hypertension and pre-eclampsia, and neonatal outcomes including hyperinsulinaemia, macrosomia, shoulder dystocia, caesarean delivery, hypoglycaemia and later life risk of obesity and T2DM [47]. Maternal implications include progression to T2DM, with ~26% and up to 70% of women with a history of GDM developing T2DM within 10–15 years of delivery [45]. GDM is also a risk factor for future maternal cardiovascular disease [48].

Universal screening for GDM is common in most developed nations [45], using a variety of tests including an oral glucose tolerance test (OGTT) at 24–28 weeks gestation for diagnosis. Selective screening is acceptable in low risk or resource poor settings [49]. Randomised controlled trials (RCTs) have demonstrated improved maternal and neonatal outcomes with subsequent treatment of GDM [50,51]. However, even with strict glycaemic control, women with GDM still have excess risks of adverse pregnancy outcomes. Although we still do not completely understand the pathophysiology, there are a range of potentially contributing factors, including chronic low-grade inflammation. Research into inflammation and biomarkers is providing important contexts and further insights into pathophysiology and risk prediction for pregnancy outcomes in GDM.

Clinical risk prediction tools for GDM are useful and have been validated in large populations [45], however sensitivity and specificity have been inadequate, and support universal screening [43]. Investigating a role of chronic low-grade inflammation in pregnancy and specifically in GDM may help enhance early prediction models to enable targeted prevention. Early identification of women at high risk of GDM may also facilitate early streamlined antenatal care with enhanced continuity, targeted lifestyle interventions to reduce GWG and potentially reduce GDM and T2DM. It may also allow timely screening and prompt GDM management, with improved patient experiences and clinical outcomes [52]. With rising GDM prevalence, opportunities for potential prevention of GDM and its complications provide rationale for early pregnancy GDM risk screening [52].

Our research group showed that an antenatal lifestyle intervention could reduce GWG and resulted in a tendency toward lower incidence of GDM in an RCT of overweight and obese women identified at high risk of GDM based on an early risk prediction tool [53]. However, the LIMIT randomised trial (Limiting weight gain in overweight and obese women during pregnancy to improve health outcomes) in Australia including over 2000 overweight and obese pregnant women found that antenatal dietary and lifestyle advice did not reduce the risk of large for gestational age (LGA) or improve maternal outcomes of pregnancy and birth, but was associated with a significant reduction in birth weight above 4000 g compared to standard care [54]. A large systematic review and meta-analysis of 182,139 obese pregnant women found reduced pre-eclampsia and shoulder dystocia in the weight management group, and reduced pre-eclampsia, gestational hypertension, preterm birth and a trend toward decreased incidence of GDM in the dietary intervention group [55]. This review was limited by heterogeneity of the individual studies, and thus individual patient data meta-analysis is now underway to provide more robust evidence for intervention effects in groups based on BMI, age, parity, socio-economic status and various medical conditions.

## 7. Inflammation and Adipokines in Pregnancies Complicated by GDM

Adipokines have provided novel links between obesity and insulin resistance, and the development of T2DM [14]. Prospective studies have shown that GDM is linked to the down-regulation of adiponectin and anti-inflammatory cytokines (e.g., IL-4 and IL-10) and up-regulation of leptin and pro-inflammatory cytokines implicated in insulin resistance (e.g., IL-6 and TNF-α) [56,57]. Altered adipokine secretion contributes to glucose homeostasis in pregnancy by both direct and indirect mechanisms: direct mechanisms include regulation of insulin secretion and insulin sensitivity; indirect mechanisms relate to inflammation, regulation of adipogenesis, chemoattraction of immune cells and subsequent effects on glucose metabolism [14].

The increase in insulin resistance in pregnancy occurs due to hormones released from the foetal-placental unit and maternal fat accretion [5]. Increased insulin resistance also relates to excess lipolysis and release of FFAs from enlarged adipose tissue, and secretion of inflammatory factors and adipokines [15]. Chronic low-grade inflammation in adipose tissue impairs insulin signalling, which further stimulates expression of genes encoding proteins implicated in insulin resistance [15]. Adipocytes synthesize substances with chemotactic and adhesive properties such as MCP-1 and vascular and intracellular adhesion molecules (VCAM, ICAM), which enhance influx of lymphocytes and monocytes [15]. Activated macrophages interact with adipocytes to initiate a perpetuating cycle of macrophage recruitment, production of inflammatory cytokines and impairment of adipocyte function, with adverse effects including insulin resistance and endothelial dysfunction [4].

Mitochondrial dysfunction due to oxidative damage has an important role in pathogenesis of chronic metabolic diseases characterized by insulin resistance, such as metabolic syndrome and T2DM [58]. The role is less well-described in GDM, but evidence has supported the role of reactive oxygen species in pathogenesis of placental insufficiency, GDM and other pregnancy complications. Pathologic pregnancies including GDM are associated with heightened levels of oxidative stress due to overproduction of free radicals (leading to abnormal mitochondrial function) and defects in antioxidant defences [59].

High sensitivity C-reactive protein (hsCRP) and sex hormone-binding globulin (SHBG) have been studied as markers for GDM. A prospective study showed that hsCRP was associated with increasing levels of maternal glucose, BMI and C-peptide [57]. Another study showed that hsCRP measured at 11–14 weeks gestation was predictive of GDM (odds ratio (OR) 3.9 for GDM development) with diagnostic specificity of 87.2%. Prospective studies have evaluated first trimester SHBG as a reliable serum marker for future risk of GDM [60,61]. The combination of low SHBG and high hsCRP had good predictive value for detection of GDM with sensitivity 74% and specificity 76% in a study of 269 women when measured before 15 weeks gestation [62].

**Figure 2:**
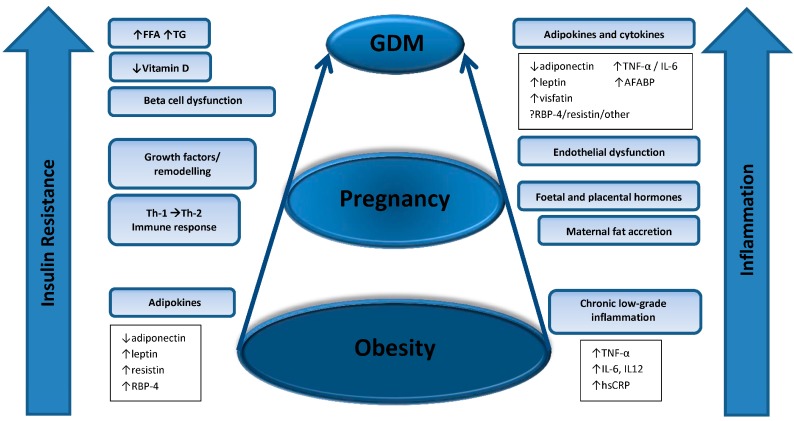
A proposed model of inflammation and insulin resistance in obesity, pregnancy and GDM. Women who are obese have features of chronic low-grade inflammation, manifest by increased tumour necrosis factor alpha (TNF-α), interleukin-6 (IL-6), interleukin-12 (IL-12), and high sensitivity C-reactive protein (hsCRP). Obesity is characterised by insulin resistance, and down-regulation of adiponectin and up-regulation of leptin, resistin and retinol-binding protein-4 (RBP4) contribute to this. Pregnancies occurring in obese women are characterised by further inflammation and a Th-2 predominant immune response, which may contribute to pregnancy complications. Foetal and placental hormones, production of abnormal growth factors and tissue remodelling may contribute to inflammation and increasing insulin resistance. GDM develops when beta cell dysfunction coexists, and may be characterised by further abnormalities in adipokine and cytokine profiles, increased free fatty acids (FFA), triglycerides (TG), low vitamin D and endothelial dysfunction.

Many adipokines and inflammatory markers affect key pathways for insulin sensitivity and secretion, however this review will focus on those thought to have a direct role in the pathogenesis of GDM. The adipokines adiponectin, leptin, TNF-α and adipocyte fatty acid-binding protein (AFABP) are increased in obesity and pregnancy and are prime candidates for direct involvement in the pathophysiology of GDM [14]. Other inflammatory markers, endothelial function markers and growth factors, and lipids will be discussed as potential predictors of GDM. Here we discuss maternal serum circulating markers, unless otherwise stated, as these have the most potential for clinical application to risk prediction tools. There are a plethora of studies of varying designs for each marker. These include many cross-sectional studies comparing prevalence of these markers in women with GDM to women without GDM, longitudinal studies (both case-control and cohort study design) evaluating predictors of GDM, and vary rarely interventional studies looking at the impact of these markers. As markers change across gestation, attempts are made to compare those measured at similar stages of gestation. Studies examine women of different ethnicities, age and BMI, use variable diagnostic criteria for GDM and various assay methods for detection of markers. Here, we will attempt to summarise the best evidence available from each marker in published studies, and Table 1 provides a summary of longitudinal studies discussed. Figure 2 illustrates the role of inflammation and insulin resistance in obesity, pregnancy and development of GDM.

**Table 1:** Longitudinal studies of inflammatory markers in prediction of GDM.

Inflammatory Marker	First Author (Year)	Study Design	GDM Status	GDM Diagnostic Criteria	Gestational Age at Testing (Weeks)	Effect in GDM	Matching or Adjustment for Confounders
TNF-α	Kirwan (2002) [63]	Prospective	5 GDM 10 NGT	24–28 weeks Carpenter and Coustan criteria [64]	Pre-gravid 12–14 weeks 34–36 weeks	TNF-α ↑ 34–36 weeks in GDM Inversely correlated with insulin sensitivity	Fat mass
	Gao (2008) [65]	Nested case-control	22 GDM 10 IGT 20 NGT	Unknown	12–20 weeks 24–32 weeks	TNF-α ↑ in GDM Positively correlated with BMI 14–20 weeks	N/A
	Georgiou (2008) [56]	Nested case-control	14 GDM 14 NGT	28 weeks ADIPS criteria 1998 [66]	11 weeks 24–28 weeks	No difference	Age ethnicity gravidity parity BMI
	Saucedo (2011) [67]	Prospective	60 GDM 60 NGT	24–28 weeks ADA criteria 2010 [ 68]	30 weeks 6 weeks 6 months postpartum	TNF-α ↑ in both groups at 6 weeks TNF-α ↑ 6months postpartum in GDM	Age weight
	Lopez-Tinoco (2012) [69]	Case-control	63 GDM 63 NGT	24–28 weeks NDDG criteria [70]	Mean ~29 weeks	TNF-α ↑ in GDM	BMI
	Guillemette (2014) [71]	Prospective	61 GDM 695 NGT	24–28 weeks IADPSG criteria [44]	5–16 weeks 24–28 weeks	TNF-α ↑ in both groups and associated with insulin resistance	Age BMI TG adiponectin.
IL-6	Morrisett (2011) [72]	Case-control	20 GDM 27 NGT	26.1 ± 3.7 weeks CDA criteria [73]	26.1 ± 3.7 weeks 8 weeks post-partum	IL-6 ↑ in GDM and post-partum, correlated with insulin sensitivity and BMI	BMI
	Hassiakos (2015) [74]	Case-control	40 GDM 94 NGT	24–28 weeks IADPSG criteria [44]	11–14 week	Il-6 ↑ in GDM and inversely related to birth weight	Maternal weight
Leptin	Kirwan (2002) [63]	Prospective	5 GDM 10 NGT	24–28 weeks Carpenter and Coustan criteria [64]	Pre-gravid 12–14 weeks 34–36 weeks	Leptin ↑ across pregnancy Inverse correlation with insulin sensitivity, but non-significant when adjusted for fat mass	Fat mass
	Georgiou (2008) [56]	Nested case-control	14 GDM 14 NGT	28 weeks ADIPS criteria 1998 [66]	11 weeks 24–28 weeks	No difference	Age ethnicity gravidity parity BMI
	Qiu (2004) [75]	Prospective	47 GDM 776 NGT	26–28 weeks Carpenter and Coustan criteria [64]	13 weeks	10-ng/mL ↑ in leptin associated with 20% ↑ GDM	Parity BMI family history of non-insulin dependent diabetes
Adiponectin	Georgiou (2008) [56]	Nested case-control	14 GDM 14 NGT	28 weeks ADIPS criteria 1998 [66]	11 weeks 24–28 weeks	Age ethnicity gravidity parity BMI	
	McManus (2014) [76]	Case-control	36 GDM 37 NGT	24–28 weeks CDA criteria [73]	31 week	Adiponectin ↓ GDM and offspring	Age maternal weight
	Williams (2004) [77]	Nested case-control	41 GDM 70 NGT	26–28 weeks Carpenter and Coustan criteria [64]	13 week	4.6-fold ↑ risk of GDM if adiponectin < 6.4 mcg/mL, overweight women 11-fold ↑ risk of GDM	BMI
	Lain (2008) [78]	Nested case-control	30 GDM 29 NGT	24–28 weeks Carpenter and Coustan criteria [64]	9.3 ± 2.6 weeks	Adiponectin < 25th 11-fold ↑ risk GDM	BMI
	Lowe (2010) [57]	Prospective	1481 pregnant women	24–32 weeks 2 h 75 g OGTT Unblinded if FPG > 5.8 mmol/L 2 h > 11.1 mmol/L or random glucose ≥ 8.9 mmol/L	24–32 weeks	↓ adiponectin associated with ↑ glucose and BMI	BMI C-peptide gestation gender
	Lacroix (2013) [79]	Prospective	38 GDM 407 NGT	24–28 weeks IADPSG criteria [44]	6–13 week	↓ adiponectin associated with ↑ risk GDM (OR1.12 per 1 µg/mL ↓ of adiponectin) and associated with insulin sensitivity	BMI HbA1c
	Ianniello (2013) [39]	Prospective	16 GDM 32 NGT	24–28 weeks Carpenter and Coustan criteria [64]	All trimesters	↓ adiponectin predictive of GDM in overweight/obese	N/A
	Weerakiet (2006) [80]	Prospective	60 GDM 299 NGT	24–28 weeks Carpenter and Coustan criteria [64]	21–27 week	Adiponectin 10 µg/mL has sensitivity of 91% and specificity of 31% for GDM	BMI
RBP-4	Krzyzanovska (2008) [81]	Nested case-control	20 GDM 22 NGT	24–28 weeks Carpenter and Coustan criteria [64]	30 week	RBP-4 ↓ in GDM	N/A
	Nanda (2013) [82]	Nested case-control	60 GDM 240 NGT	24–28 weeks WHO criteria 2006 [83]	11–13 weeks	No difference	N/A
	Abetew (2013) [84]	Nested case-control	173 GDM 187 NGT	24–28 weeks Carpenter and Coustan criteria [64]	16 weeks	RBP-4 ↑ in GDM but not significant after adjustment	Maternal age ethnicity
Resistin	Lain (2008) [78]	Nested case-control	30 GDM 29 NGT	24–28 weeks Carpenter and Coustan criteria [64]	9.3 ± 2.6 weeks	No difference	BMI
	Georgiou (2008) [56]	Nested case-control	14 GDM 14 NGT	28 weeks ADIPS criteria 1998 [66]	11 weeks 24–28 weeks	No difference	Age ethnicity gravidity parity BMI
	Lowe (2010) [57]	Prospective	1481 pregnant women	24–32 weeks 2 h 75 g OGTT Unblinded if FPG > 5.8 mmol/L 2 h > 11.1 mmol/L or random glucose ≥ 8.9 mmol/L	24–32 weeks	Not associated with glucose or birth weight	BMI C-peptide gestation gender
	Nanda (2012) [85]	Nested case-control	60 GDM 240 NGT	24–28 weeks WHO criteria 2006 [83]	11–13 weeks	No difference	N/A
	McManus (2014) [76]	Case-control	36 GDM 37 NGT	24–28 weeks CDA criteria [73]	31 weeks	Resistin ↓ in GDM and offspring	Age maternal weight
Visfatin	Krzyzanovska (2006) [86]	Nested case-control	64 GDM 30 NGT	24–28 weeks Carpenter and Coustan criteria [64]	28–30 weeks 38–40 weeks 2 weeks post-partum	Visfatin ↑ in GDM	BMI
	Ferreira (2011) [87]	Case-control	100 GDM 300 NGT	24–28 weeks WHO criteria 2006 [83]	11–13 week	Visfatin ↑ in GDM	N/A

NGT = normal glucose tolerance; IGT = impaired glucose tolerance; FPG = fasting plasma glucose; HOMA-IR = homeostasis model assessment for insulin resistance; Matsuda index = measure of insulin sensitivity; BMI = body mass index; TG = triglycerides; N/A = not available; ↓ = decreased levels; ↑ = increased levels; ADIPS = Australasian Diabetes in Pregnancy Society; ADA = American Diabetes Association; NDDG = National Diabetes Data Group; IADPSG = International Association of Diabetes and Pregnancy Study Group; CDA = Canadian Diabetes Association; WHO = World Health Organisation.

### 7.1. Tumour Necrosis Factor-α (TNF-α) and Interleukin-6 (IL-6)

TNF-α and IL-6 are produced by adipose tissue monocytes and macrophages and lead to insulin resistance [14]. Placental production of these cytokines contributes to pregnancy related insulin resistance [5]. TNF-α impairs insulin signalling and beta cell function, which may directly contribute to GDM [14]. Further, increased levels of TNF-α and IL-6 occur due to oxidative stress and inflammatory changes induced by hyperglycaemia such as in GDM [5]. TNF-α mRNA and protein expression in adipose tissue correlate positively with adiposity, and decrease in obese individuals after weight loss [14]. Consistent evidence shows up-regulation of TNF-α in GDM, however it remains controversial as to whether up-regulation precedes or is a consequence of disease. Most cross-sectional studies show that circulating TNF-α concentrations are increased in the second and third trimesters [14], correlate with pre-pregnancy BMI [88,89] and predict insulin resistance and GDM [14,88,90]. However, these studies are limited by small sample sizes, and most fail to adjust for BMI. A case-control study found that plasma TNF-α was significantly higher in women with GDM [69], but a smaller study failed to replicate these findings [56]. A meta-analysis of 10 observational studies found significantly elevated TNF-α in serum of GDM *vs.* normal pregnancies, which persisted in a sub-analysis where GDM patients were compared to BMI matched controls [91]. One prospective study demonstrated an association between TNF-α levels and insulin resistance in the first and second trimesters when adjusted for age, BMI, triglycerides (TG) and other confounders [71]. Another prospective study of 120 women found increased homeostasis model assessment of insulin resistance (HOMA-IR) in women who developed GDM compared to those with normal glucose tolerance (NGT), but no difference in TNF-α [67]. In a study of 15 women using euglycaemic-hyperinsulinaemic clamps, TNF-α was increased in late pregnancy. TNF-α was higher in women who developed GDM (*n =* 5) compared to those with normal glucose tolerance (*n =* 10), after adjustment for fat mass. Furthermore, TNF-α was inversely correlated with insulin sensitivity on clamp [63]. A fourth prospective study found a significant association between TNF-α levels and GDM in an Asian cohort [65]. Differences in these studies may relate to use of different diagnostic criteria for GDM, differing ethnicities, small sample size and variable matching and adjustment for confounders, particularly BMI (see Table 1). Although promising, more research is needed to clarify the role of TNF-α as a predictor of GDM development independent of BMI.

IL-6 is over-expressed in obesity and inflammation [24]. A rise in IL-6 in pregnancy, principally due to placental production, has been linked to pregnancy related insulin resistance [5]. IL-6 is also up-regulated in women with GDM at delivery [90]. In cross-sectional studies, IL-6 concentration positively correlated with percent body fat, BMI, insulin sensitivity and plasma glucose levels during pregnancy and after delivery [24,72,92]. In case-control studies, plasma IL-6 levels are increased in GDM independently of obesity [72], and may be a significant predictor of GDM (see Table 1) [74]. However, to our knowledge, there are no prospective studies confirming this.

### 7.2. Leptin

Leptin is a protein hormone that plays an important role in the regulation of whole body metabolism [17]. It has been shown to influence insulin secretion, glucose utilisation, glycogen synthesis and fatty acid metabolism [5,42]. Leptin is released into the circulation by adipose tissue in proportion to lipid stores [5].

Obesity and pregnancy are leptin resistant states associated with impaired leptin signalling in the hypothalamus [42]. In clinical studies, serum leptin concentrations are directly proportional to fat mass, and decreased central leptin responsiveness or leptin resistance is seen in obesity [14]. Maternal leptin levels increase from the earliest stages of pregnancy, implying that the increases are not only due to maternal weight gain [42]. The placenta also expresses high amounts of leptin messenger RNA and protein, leptin receptors are abundant in the placenta [5], and leptin secreted from the placenta may contribute to regulation of foetal growth independent of maternal glucose levels [14].

Leptin may contribute to GDM pathophysiology by suppressing insulin secretion from pancreatic beta cells [14]. Other effects of leptin related to appetite control, body weight and composition and energy expenditure via effects on the hypothalamus are involved in pathogenesis of GDM [14]. Increased leptin synthesis in GDM amplifies low-grade inflammation by stimulating production of pro-inflammatory cytokines such as IL-6 and TNF-α, which further enhances leptin production [42].

Cross-sectional studies have described increased circulating concentrations of leptin in women with GDM [69,90,93]. A meta-analysis including 18 observational studies found that leptin concentrations were significantly higher in GDM patients compared to controls, and remained elevated when compared to BMI matched controls [91]. A small nested case-control study, screening for biomarkers predictive of GDM in the first trimester did not find an association with leptin [56]. However, a larger prospective cohort study by Qiu *et al.* [75] found that hyperleptinaemia at <16 weeks gestation was predictive of increased risk of GDM. There was a strong linear correlation, with each 10 ng/mL increase in leptin concentration associated with a 20% increase in GDM risk, independent of maternal pre-pregnancy adiposity and other confounders (see Table 1) [75]. In a clamp study of 15 subjects (5 with GDM) by Kirwan *et al.* [63], circulating leptin levels increased from pregravid to early pregnancy, and remained elevated throughout late pregnancy. Leptin levels were lower in lean women with NGT compared to obese women with GDM, and were inversely correlated with insulin sensitivity. However, the correlation was no longer significant when adjusted for fat mass [63]. A recent meta-analysis evaluating eight prospective studies found that leptin levels in the first or early second trimester were significantly higher (7.25 ng/mL) in women who later developed GDM compared to those who did not [94]. They reported no significant heterogeneity between studies in regards to timing of blood collection, assay method, or diagnostic criteria for GDM [94]. However, the assessment of BMI and adiposity varied, and no conclusions were drawn regarding the role of leptin independent of adiposity for prediction of GDM. In summary, leptin appears to have a role in inflammation and pathophysiology of GDM. However, studies have not adequately addressed the confounding influence of BMI/adiposity and gestational weight gain on leptin levels. Thus further prospective studies are required to determine predictive ability in GDM.

### 7.3. Adiponectin

Adiponectin is an abundant plasma protein secreted exclusively from adipose tissue and is decreased in obesity [17]. Adiponectin circulates in the serum as a range of multimers and the high molecular weight (HMW) isoform is the most active form, accounting for the majority of its peripheral metabolic effects [17]. Adiponectin is an insulin-sensitizing, anti-inflammatory and anti-atherogenic adipokine that stimulates glucose uptake in skeletal muscle and reduces hepatic glucose production through AMP-activated protein kinases [26,42,95]. In clinical studies, circulating adiponectin is independently and negatively related to features of the metabolic syndrome such as insulin resistance, bodyweight, blood pressure and serum lipids [14,96].

In normal pregnancy, maternal adiponectin secretion progressively declines, and levels negatively correlate with BMI and adiposity [17]. Hypoadiponectinaemia exacerbates insulin resistance and correlates with β cell dysfunction [97], the hallmarks of GDM. Adiponectin mRNA is also down-regulated in placental tissue in women with GDM [42]. Furthermore, it is thought that TNF-α and other pro-inflammatory mediators secreted in GDM suppress the transcription of adiponectin by adipocytes [42], further aggravating chronic low-grade inflammation.

A recent systematic review and meta-analysis of 15 cross-sectional and case-control studies [91] found a significantly lower adiponectin level in GDM patients compared to controls, which remained significantly lower in GDM patients compared to their BMI matched controls. A further meta-analysis of nine prospective studies showed consistently that adiponectin levels in the first and second trimester were lower in women who later developed GDM than those who did not [94].

Down-regulation of adiponectin may predict GDM several months before clinical diagnosis, independent of BMI status [56,76,77,78] and insulin sensitivity [56,77,78]. For example, Williams *et al.* found that plasma adiponectin concentrations <6.4 µg/mL compared to higher concentrations at 13 weeks gestation increased risk of GDM by 4.6-fold [77]. These findings have been validated in prospective studies (see Table 1) [39,57,79,80]. In a prospective cohort study, women with lower first trimester adiponectin had increased risk of developing GDM even after adjustment for BMI and first trimester haemoglobin A1c (HbA1c) (OR 1.12 per 1 µg/mL decrease of adiponectin, *p* = 0.02) [79]. Furthermore, adiponectin levels in the first and second trimesters were strongly associated with HOMA-IR and Matsuda Index for insulin sensitivity [79].

Evidence is also emerging that maternal adiponectin decreases foetal growth by impairing placental insulin signalling and reducing insulin-stimulated amino acid transport [14]. Decreased concentrations may contribute to foetal macrosomia in women with GDM [98] and in women without GDM [99]. Hypoadiponectinaemia persists post-partum after GDM, and may contribute to progression to T2DM [3,100,101].

A large prospective study by the HAPO investigators looked at the association of inflammatory mediators with maternal glucose and birth size, adjusted for maternal BMI, fasting C-peptide and other potential confounders (see Table 1) [57]. They reported that mean levels of adiponectin were lower, and hsCRP was higher across increasing levels of maternal glucose, BMI and C-peptide. Adiponectin and hsCRP were inversely associated with birth weight, neonatal sum of skinfolds and percent body fat after adjustment [57].

In summary, there is good evidence that adiponectin is lower in obesity, in pregnancy and in GDM. It appears to be involved in the pathophysiology of GDM, and is predictive of risk for GDM. Studying the impact of interventions such as lifestyle or metformin on regulation of adiponectin will be important to further understand how chronic low grade inflammation contributes to insulin resistance in GDM.

### 7.4. Adipocyte Fatty Acid-Binding Protein (AFABP)

Adipocyte fatty acid-binding protein (AFABP) belongs to the fatty-acid binding protein family and is highly expressed in adipocytes, macrophages and endothelial cells [14]. High circulating levels have been found to independently predict risk of metabolic syndrome, T2DM and cardiovascular disease [102]. Serum levels or AFAPB are increased in overweight and obese subjects compared to lean controls and correlate positively with waist circumference, blood pressure and insulin resistance [102]. Furthermore, levels may be predictive of T2DM, independent of obesity, insulin resistance or glycaemic indices [102]. Serum concentrations of AFABP were increased in the third trimester in a cross-sectional study of women with GDM compared to controls matched for gestational age and insulin sensitivity [103]. GDM was independently associated with AFABP concentration, and markers of the metabolic syndrome including leptin, BMI and triglycerides (TG) were significantly associated with serum AFABP concentrations [103]. There is good evidence that AFABP is up-regulated in GDM after adjustment for pre-pregnancy BMI, and a further cross-sectional study found associations with newborn size and adiposity [104]. However, thus far there are no prospective studies of AFABP in prediction of GDM.

### 7.5. Retinol-Binding Protein-4 (RBP-4)

Retinol-binding protein-4 (RBP-4) is a blood carrier protein for retinol synthesized in hepatocytes and adipocytes [97]. Increased circulating levels have been reported in several metabolic complications including obesity, insulin resistance, polycystic ovary syndrome and cardiovascular disease [42]. Evidence for association with pregnancy complications remains inconclusive as there are no consistent results on RBP-4 regulation in normal pregnancy and cross-sectional studies of GDM are contradictory reporting increased, decreased and unaltered plasma levels [97]. This may relate to the strong associations of RBP-4, BMI and insulin resistance [42]. In addition, RBP-4 binds to tissue transthyretin (TTR) *in vivo*, and higher circulating TTR concentrations have been found in glucose-intolerant women with previous GDM [82], resulting in increased formation of an RBP-4-TTR complexes and reduced RBP-4 clearance. A large meta-analysis of observational studies found that maternal circulating RBP-4 levels were significantly higher in GDM patients than controls, however this difference was limited to Asian women [105]. Evidence thus far does not support a role of RBP-4 for prediction of GDM, with two nested case-control studies showing that RBP-4 does not predict risk of GDM when tested in the first trimester [82,84], and another showing that RBP-4 levels are actually reduced in GDM (see Table 1) [81].

### 7.6. Resistin

Resistin is a hormone expressed abundantly in monocytes and macrophages, and to a lesser extent adipocytes [5]. It may have a role in inducing inflammation, endothelial dysfunction, thrombosis, angiogenesis and smooth muscle dysfunction [14]. We have previously shown that plasma resistin is related to adiposity but not insulin resistance measured by glucose clamp in healthy humans [106]. Plasma resistin levels are higher in pregnancy likely due to weight gain and increased adiposity, and circulating levels are thought to increase with advancing gestation with progressive weight gain [42]. Placental expression of resistin is up-regulated in the third trimester [5]. Resistin is thought to impair glucose tolerance in pregnancy and several studies have shown a positive correlation between obesity and insulin resistance in pregnancy and elevated plasma resistin, but others have not found this association [97]. Most case-control studies have found no difference in resistin levels in women with GDM [56,78,85], confirmed in a large prospective study [57]. However, other studies have shown increased and decreased levels [14,76,92]. Despite increased levels in GDM, Kuzmicki *et al.* were unable to demonstrate an association between serum resistin and insulin levels or insulin resistance [92]. A recent meta-analysis of 10 studies found no difference in plasma resistin level between women with GDM and pregnant controls [107]. However, there was considerable heterogeneity in the analysed studies including wide variations in mean resistin concentration in both GDM and controls [107]. Three prospective studies have shown that resistin does not contribute to risk prediction of GDM when adjusted for BMI (see Table 1) [56,78,85]. Overall it appears that resistin may mediate insulin resistance during pregnancy, but it is unlikely to have a central role in glucose homeostasis and development of GDM [3].

### 7.7. Visfatin

Visfatin is highly expressed in visceral adipose tissue, promotes adipogenesis and exerts insulin-mimetic effects [5]. Visfatin may also up-regulate production of pro-inflammatory cytokines by monocytes [108]. Circulating levels are thought to increase in obesity and insulin resistant states [5], and elevated visfatin levels have been shown in T2DM [97]. Visfatin may improve insulin sensitivity during the second and third trimesters and up-regulation in insulin resistance associated pregnancy complications may be part of a physiological feedback mechanism to improve insulin signalling [5]. A recent study found a 7-fold increase in visfatin gene expression and protein in omental fat of pregnant women compared to controls, but only a small increase in serum levels, suggesting its role may be more paracrine than as a hormone [5]. Visfatin is reported to be both decreased and increased in GDM. Lewandowski *et al.* [109] found positive correlations of plasma visfatin with fasting and post-glucose load insulin in women with GDM in the third trimester in a cross-sectional study. In a nested case-control study, Krzyzanowska *et al.* [86] reported higher plasma visfatin in GDM women compared to controls with normal glucose tolerance, but there was no relationship with fasting plasma glucose, insulin, insulin resistance or BMI. Ferreira *et al*. [87 found an increased level of visfatin in the first trimester of women who later developed GDM, suggesting that it could be a potential biomarker for GDM (see Table 1). However, further studies are required to evaluate the relationship with obesity, and any causal association with insulin resistance and GDM.

In summary, decreased adiponectin is an independent predictor of GDM. Increased TNF-α and leptin may also be predictive, but studies must firmly establish their role independent of BMI and insulin resistance. Preliminary evidence suggests IL-6, AFABP and visfatin may be predictive of GDM, but prospective studies are required. Although RBP-4 and resistin have been associated with obesity and insulin resistance, and may be altered in pregnancy, they do not appear to be predictive of GDM development.

### 7.8. Novel Adipokines

Vaspin (visceral adipose tissue-derived serpin A12) is a member of the serine protease family expressed in visceral adipose tissue with insulin sensitising properties [42]. The role of vaspin in pregnancy is not well understood and levels are reported to be both increased and decreased [110]. Vaspin levels were higher in GDM and positively correlated to leptin, HOMA-IR and TG levels compared to age matched women with NGT and non-pregnant women [111]. However, in two more recent studies vaspin levels were not significantly altered in GDM and were not associated with markers of insulin resistance in pregnant patients [112,113].

Apelin is an angiogenic factor and adipokine implicated in glucose homeostasis [5]. Insulin and TNF-α exert direct control on apelin gene expression in adipocytes [5]. Apelin has been found to be increased in obese individuals and T2DM [14]. Cross-sectional studies of circulating apelin in GDM have contradictory results including unaltered and increased levels [14].

Omentin is an adipokine produced by visceral fat that has been linked to susceptibility to T2DM [14,97]. Omentin has been shown to be higher in the first trimester of pregnancy than in the second, suggesting either increased clearance or reduced secretion [97]. Maternal obesity was associated with lower omentin in plasma, adipose tissue and the placenta and negatively correlated with birth weight in one case-control study [114]. Maternal omentin was significantly lower in non-obese GDM women compared to non-obese NGT women, but no different between obese GDM and obese NGT women [114]. Thus far, these novel adipokines have not been studied prospectively for prediction of GDM.

## 8. Other Potential Biomarkers in Pregnancies Complicated by GDM

### 8.1. Endothelial Function and Angiogenic Growth Factors

Mordwinkin *et al.* [115] demonstrated the presence of decreased maternal circulating endothelial progenitor cells, increased soluble adhesion molecules in maternal blood, decreased expression of superoxide dismutase in maternal and cord blood and increased endothelial nitric oxide synthase (NOS) expression in maternal and cord blood of women with GDM. These findings were consistent with mechanisms where hyperglycaemia leads to increased oxidative stress and endothelial dysfunction in GDM mothers and their foetuses.

Lappas *et al.* found increased expression of angiogenic proteins and adhesion molecules in omental adipose tissue from women with GDM and pre-existing obesity at Caesarean section [116]. Pre-existing maternal obesity and GDM were associated with increased gene expression of placental growth factor (PLGF), soluble endoglin (sEng) and intracellular adhesion molecule-1 (ICAM-1) and increased secretion of PLGF, soluble fms-like tyrosine kinase-1 (sFlt-1), fibroblast growth factor-2 (FGF2), sEng and sICAM-1 [116]. Markers related to endothelial function and angiogenesis found to be raised in GDM after adjustment for confounders including obesity include tissue plasminogen activator (TPA) [117], fibroblast growth factor-21 (FGF-21) [118] and glycosylated fibronectin [119]. In the prospective HAPO study, plasminogen activator inhibitor-1 (PAI-1) increased across increasing levels of maternal glucose, BMI and C-peptide, and was also associated with sum of skinfolds in the neonate [57]. First trimester follistatin-like-3 has been found to be decreased in women with later development of GDM [120]. These findings need to be validated in further prospective studies.

### 8.2. Vitamin D

Low vitamin D has been implicated in the aetiology of obesity, insulin resistance and T2DM [121]. One of the main mechanisms involved may be chronic low-grade inflammation [121]. Vitamin D deficiency is common in pregnancy, and may contribute to abnormal glycaemic regulation [122]. Early pregnancy vitamin D status has been inversely associated with GDM risk [123]. However, Kramer *et al.* found that increased parathyroid hormone, rather than vitamin D deficiency was independently associated with dysglycaemia in pregnancy in 524 women when tested in conjunction with the OGTT [124]. In a large prospective cohort of pregnant women (*n =* 655 with 54 who developed GDM), Lacroix *et al.* [122] found lower first trimester 25-hydroxyvitamin D (25-OHD) levels were associated with higher risk of GDM after adjustment for vitamin D confounders and GDM risk factors. Lower first trimester 25-OHD was also associated with markers of insulin resistance in the second trimester [122]. However, another prospective study of first trimester 25-OHD in 248 women did not find evidence of an association with GDM development, although 25-OHD level correlated with 2 h OGTT, high density lipoprotein cholesterol (HDL), ethnicity, obesity and smoking [125]. Three recent systematic reviews [126,127,128] concluded that 25-OHD deficiency was associated with higher risk of GDM. However, the reviews were limited by the observational nature of the included studies and important confounders such as ethnicity and adiposity [129].

A randomised controlled trial (RCT) of 56 women with GDM diagnosed at 24–28 weeks gestation randomised to calcium plus 25-OHD (50,000 international units (IU) at baseline and day 21) compared to placebo, found a significant reduction in fasting glucose, serum insulin levels, HOMA-IR, and a significant increase in the quantitative insulin sensitivity index in the intervention group [130]. Another RCT of women with 25-OHD levels <80 nmol/L at mean 14 weeks gestation randomised to high dose 25-OHD (5000 IU daily) *vs.* routine pregnancy dosages (400 IU daily) until delivery, found no difference in maternal glucose levels on OGTT [131]. However, many women (34% of low dose and 10% of high dose group) remained 25-OHD deficient after therapy. Further RCTs are crucial to determining whether vitamin D has a role in pathophysiology of GDM and whether supplementation has a role in preventing GDM [129].

### 8.3. Lipid Metabolism

Dyslipidaemia is well established in obesity. In pregnancy, accumulation of maternal fat deposits and hyperlipidaemia occurs. Although maternal triglycerides are unable to directly cross the placenta, diffusion of fatty acids to the foetus is ensured by the presence of lipoprotein receptors, lipoprotein lipase activity and intracellular lipase activity in the placenta [132]. It is thought that fatty acids may contribute to foetal growth and increased fat mass [133]. Maternal plasma triacylglycerols (TAG) and non-esterified fatty acids (NEFA) correlate with foetal lipids, foetal growth and fat mass [133]. In women with GDM, there is a positive correlation between maternal TAG concentrations and neonatal body weight or fat mass even in normoglycaemic conditions [133]. This is notable in the context that more glucose crosses the placenta than any other substrate, however correlations between maternal glucose concentrations and foetal growth are not consistent [133].

Alterations in lipid metabolism may also be predictive of GDM. A cross-sectional study found that triglycerides (TG) and atherogenic indices measured at 24–28 weeks were higher in women who developed GDM [134]. In a larger case control study, those that developed GDM had decreased first trimester HDL after adjustment for confounders including BMI [117]. However, a prospective study found that TG measured in the first and second trimester did not differ significantly in women with GDM or insulin resistance after adjustment for BMI, HbA1c, age and Matsuda Index for insulin sensitivity [79]. Lipoprotein (a) was not associated with insulin sensitivity and GDM in a small study [135]. The role of altered lipid metabolism in early pregnancy for prediction of GDM independent of BMI and insulin resistant phenotypes is unproven, and requires more longitudinal research.

Di Cianni *et al.* [136] studied 83 women with an abnormal glucose challenge but normal OGTT and found that pre-pregnancy BMI and fasting maternal serum TG (>75th percentile) in the third trimester were independently associated with neonatal birth weight. Schaefer-Graf *et al.* [137] performed a prospective study of 150 women looking at maternal lipids including serum TG, cholesterol, free fatty acids (FFA), glycerol, insulin and glucose in maternal serum and cord blood during the third trimester. Maternal TG and FFA measured at 28 weeks correlated well with foetal abdominal circumference, and at delivery correlated with anthropometric measures [137]. After adjustment for confounders including maternal BMI, maternal FFA and TG were independent predictors of large for gestational age neonates [137]. Prospective studies provide compelling evidence that altered lipid metabolism, in particular serum TG and FFA in late pregnancy, are strong predictors of neonatal birth weight. Further research is required to assess whether these effects can be diminished or prevented by reducing TG and FFA, for example by an antenatal dietary intervention.

### 8.4. Metabolomics

Metabolomics using sensitive techniques such as liquid chromatography–mass spectrometry (LC–MS), has been used to construct metabolic profiles and identify novel pathways in T2DM [138]. Recently this technique has been applied to metabolite profiling in GDM. Whilst profiling has yielded inconsistent results due to small patient populations, differing methodology in analysis, and variations in glycaemic control or treatment among participants, a recent systematic review found that the biomarkers most consistently associated with GDM were asymmetric dimethylarginine (ADMA, a metabolic product of protein modification in the cell cytoplasm with potential roles in endothelial dysfunction, insulin resistance and cardiometabolic diseases) and NEFAs (the major components of TAGs, initially described in studies of insulin resistance and T2DM) [138]. Recent metabolomics studies have attempted to determine biomarkers for diagnosing GDM at 14–25 weeks gestation, with inconsistent findings [138]. The benefits of metabolomics in GDM need further investigation in larger more diverse populations [138].

## 9. Limitations of Literature on Inflammatory and Other Markers in GDM

Despite the growing number of studies on inflammatory and other markers in GDM, the literature is largely inadequate. Variations in the populations studied, and GDM diagnostic criteria applied makes comparison of studies difficult. In many cases studies are cross-sectional or case-control design with small sample sizes and thus are largely hypothesis generating. In addition, studies available often neglect to adjust for important confounders including maternal age, ethnicity, smoking status, BMI, GWG and glucose levels. The other major limitation in this novel area of research is in the use of different types of samples (serum, plasma or culture supernatant), sample source (maternal, placental, cord) and different assay methods (e.g., Enzyme-linked immunosorbent assays, chemiluminescent immunoassay, immunoradiometric assay) [40]. However, even when the same assay methods are utilised, there is variability due to lack of standardised measures and reporting. Studies need to provide more detailed information on sample collection, handling, storage and assessment methods, and report findings according to internationally accepted standards [40]. There are also little prospective data and very limited interventional studies that explore the relationships between these markers and GDM. Better designed prospective studies with larger populations are required to explore pathophysiology, confirm a predictive role of inflammatory markers and to provide insight into potential future treatments and prevention.

## 10. Risk Prediction Models and Their Clinical Application: Is There a Role for the Addition of Biomarkers?

Multi-parametric risk prediction models combining inflammatory and other biomarkers with maternal clinical risk factors may enhance prediction tools for GDM. This is particularly important in first pregnancies. An effective predictive model for risk of GDM may incorporate clinical risk factors and biomarkers (indicative of inflammation or insulin resistance) that precede the onset of hyperglycaemia, potentially avoiding harm caused by overt glucose intolerance in late pregnancy [43]. Early detection of women at risk of GDM would allow streamlined antenatal care, institution of an appropriate care model and level of clinical surveillance, with enhanced continuity of care and prompt management of GDM where it occurs [52]. Early prediction would also allow targeted dietary and lifestyle interventions to reduce GWG and development of GDM [52], and improve pregnancy outcomes [139,140].

There are a number of published clinical risk prediction tools that have been validated, achieving good sensitivity and specificity for prediction of GDM [141]. This includes a tool developed by our team and validated internationally, which aimed to identify women at risk of GDM and trial an antenatal lifestyle intervention to prevent GDM [45]. A simple risk prediction tool based on previous GDM, family history of T2DM, high risk ethnicity, age and BMI, achieved a sensitivity of 61.3% and specificity of 71.4% for differentiating women according to their risk of GDM [45]. Notably, the model achieved even better performance for identifying women with GDM requiring insulin, with an area under the receiver operating characteristic curve (AUC) of 0.74 for identifying women with GDM and an AUC 0.81 for women with GDM who required insulin [141]. These high risk women may accrue the most benefit from early prediction and intervention. We progressed this work in those identified at high GDM risk in the clinical prediction tool, measured and examined fasting biochemical markers (glucose, lipids) at 12–15 weeks gestation and GDM diagnosis at 28 weeks gestation in our cohort [52]. We classified women with GDM based on Australasian Diabetes in Pregnancy Society (ADIPS) criteria (prevalence 23%) and IADPSG criteria (30%). Fasting glucose at 14–16 weeks was the strongest predictor for GDM and incrementally improved the tool with sensitivity and specificity of 97% and 94.8% respectively for ADIPS-diagnosed GDM (AUC 0.79) and 94% and 92.4% for IADPSG-diagnosed GDM (AUC 0.83) [52].

Overall, adding inflammatory and other biomarkers to clinical GDM risk prediction tools has shown little promise to date. While incremental sensitivity and specificity is seen by addition of inflammatory and other biomarkers to clinical tools for GDM [74,87,117,142], translation to clinically important improvements in prediction is debatable, with very few implementation studies performed. The challenge is to find a tool which improves accuracy, is clinically feasible, affordable and convenient [52]. When studied in isolation, none of the markers reviewed here provide adequate positive predictive value, and thus combinations of markers have the most potential application if feasible and cost-effective. However, we would suggest that with reasonably accurate and simple clinical prediction models already available, the incremental benefit for future risk prediction is limited, and it is unlikely to be cost-effective. Rather, the interest in the markers reviewed here will mainly be in the insight they may provide into pathophysiology and potential future prevention and treatment of GDM and related pregnancy complications.

## 11. Conclusions

Obesity is increasing in prevalence, and is characterised by a pro-inflammatory and insulin resistant state. Pregnancy induces an inflammatory state, and worsening of insulin resistance during pregnancy is further exacerbated by obesity and gestational weight gain, and may result in GDM (see Figure 2). GDM is increasingly common, and obesity and GDM have adverse impacts on short and long term maternal and child health. Currently, the pathophysiology of GDM and related adverse health outcomes remains unclear. An extensive body of research is available on inflammatory and other potential markers that may relate to GDM and provide insights into pathophysiology. The imbalance in expression of pro-inflammatory and anti-inflammatory markers may contribute directly to impaired glucose homeostasis. Yet, current evidence in this field still has significant gaps related to variable GDM diagnostic criteria and a diversity of methods used for measurement of markers. Also, there are limitations in study design with most studies being cross-sectional studies looking at relationships between these markers and GDM status. There are few prospective studies confirming markers that independently predict GDM, and no readily identified intervention studies looking at modulation of these markers and impact on GDM risk, with further research clearly needed.

The clinical utility of risk prediction tools depends on patient and clinician acceptability, cost-effectiveness, the ability to distinguish low and high risk pregnancies, and demonstration of clinically meaningful reductions in adverse outcomes [2]. Use of predictive tools has provided important contributions to maternal-foetal medicine for prediction of adverse outcomes such as aneuploidy [2] and hypertensive disorders [143]. There is emerging evidence that the 11- to 13-week assessment is likely to be the basis for a new approach to antenatal care, whereby data from the maternal history will be combined with the results of biophysical and biochemical tests to estimate the patient-specific risk for a wide variety of pregnancy complications [38]. In the case of GDM, there are clinically useful tools available for early prediction. It is arguable whether inflammatory and other biomarkers will improve on existing risk prediction tools in a feasible, practical and cost-effective way. We suggest that the future of research into biomarkers and GDM is best focused on overcoming methodological limitations of existing studies, providing insights into pathophysiology of GDM and related complications and in suggesting potential strategies to prevent GDM or reduce the severity of glucose intolerance, and prevent adverse pregnancy outcomes.
